# Causal effects of specific gut microbiota on spinal stenosis diseases: a two-sample mendelian randomization study

**DOI:** 10.3389/fgene.2024.1400847

**Published:** 2024-09-26

**Authors:** Kaihang Luo, Weizheng Zeng, Qiushuang Li, Yuliang Zhang, Shengkai Liu, Xizhe Liu, Shaoyu Liu

**Affiliations:** ^1^ Department of Spinal Surgery, The First Affiliated Hospital, Sun Yat-sen University, Guangzhou, China; ^2^ Department of Obstetrics and Gynecology, Guangdong Provincial Key Laboratory of Major Obstetric Diseases, Guangdong Provincial Clinical Research Center for Obstetrics and Gynecology, Guangdong-Hong Kong-Macao Greater Bay Area Higher Education Joint Laboratory of Maternal-Fetal Medicine, Guangzhou, China

**Keywords:** gut microbiota, adolescent idiopathic scoliosis, lumbar spondylolisthesis, spinal stenosis, mendelian randomization

## Abstract

**Background:**

Although recent observational studies and clinical trials have indicated a strong association between the gut microbiota and spinal stenosis diseases, the causal relationship between them remains unclear.

**Methods:**

Based on large-scale genome-wide association studies, we employed two-sample Mendelian randomization (MR) to analyse the causal relationships between the gut microbiota (GM) and 3 spinal stenosis diseases: adolescent idiopathic scoliosis (AIS), lumbar spondylolisthesis (LS), and spinal stenosis (SS). MR analysis was performed using the inverse variance weighting (IVW) method as the primary approach, supplemented by MR‒Egger regression, weighted median, and weighted mode analyses. MR-PRESSO and MR‒Egger regression were employed to assess horizontal pleiotropy. Cochran’s Q test was used to evaluate heterogeneity. Further leave-one-out sensitivity analysis was conducted to ascertain the reliability of the causal relationships.

**Results:**

The IVW method identified 9 gut microbiota taxa (9 genera) that were causally related to AIS, 14 taxa (4 phyla, 2 classes, 2 orders, 1 family, and 5 genera) to LS, and 4 taxa (2 classes, 1 order, and 1 genus) to SS. The Cochrane Q test results did not indicate heterogeneity. Moreover, both the MR‒Egger intercept test and the MR-PRESSO global test demonstrated that our findings were robust against potential horizontal pleiotropy. Furthermore, leave-one-out analysis provided additional evidence supporting the reliability of our identified causal relationships.

**Conclusion:**

Our findings have substantiated the potential causal impact of specific GM taxa on AIS, LS, and SS, thereby offering novel insights into the mechanisms mediated by the gut microbiota in these three diseases and laying the foundation for targeted preventive measures in further research.

## 1 Introduction

As the global population ages, the incidence of low back pain (LBP) is escalating. Spinal stenosis disease (SSD) stands out as a major contributor to LBP, affecting an estimated 11% of the global population (Jensen et al., 2020). SSD is characterized by a constriction of the lumbar spinal canal and neural foramina, which can lead to compression of the spinal cord and encroachment upon adjacent nerve roots. SSD presents in both congenital and acquired forms, with the latter primarily attributed to factors such as intervertebral disc herniation, scoliosis, spondylolisthesis, ligament hypertrophy, and the development of osteophytes in the facet joints. Clinical presentations include lower back pain, neurogenic claudication, radicular pain, and lower extremity weakness. In the United States in 2014, over 350,000 patients aged 45 or older underwent laminectomy, with an additional 370,000 individuals undergoing fusion surgeries, the majority of which were attributable to SSD (Hoffman et al., 2021). Patients are confronted not only with the substantial financial burden of surgery but also with the physical and psychological consequences inherent in these procedures.

Scoliosis, characterized by its multifaceted three-dimensional spinal deformity, is a condition that manifests with a particular prevalence during adolescence. Adolescent idiopathic scoliosis (AIS) is recognized as the most common form of scoliosis affecting children within the age bracket of 11–18 years. The global prevalence of scoliosis ranges from 0.47% to 5.20% (Konieczny et al., 2013), while in the Chinese population, it is estimated to be between 0.6% and 2.0%, with AIS constituting approximately 90% of all cases (Qiu, 2017). Untreated AIS can lead to a spectrum of long-term sequelae that encompass not only physical manifestations, such as the progression of spinal curvature, back pain, and potential cardiopulmonary complications, but also significant psychosocial challenges for the individuals affected. Patients with a Cobb angle of less than 30° at skeletal maturity are at low risk for further progression. In cases with a Cobb angle between 30° and 50°, an average progression of 10°–15° is observed over a lifetime. However, Cobb angle over 50° at maturity are prone to a steady progression at a rate of approximately 1° per year. Notably, life-threatening effects on pulmonary function typically do not manifest until the scoliotic curve reaches 100° or greater (Reamy and Slakey, 2001).

Lumbar spondylolisthesis (LS), characterized by the anterior or posterior displacement of one vertebra onto another, can be categorized into distinct etiological subtypes: isthmic, degenerative, dysplastic, pathological, and traumatic (Koslosky and Gendelberg, 2020). Spondylolysis is the most common cause in children and adolescents, with a prevalence ranging from 3% to 10% in the population, and approximately 70% may progress to spondylolisthesis (Foreman et al., 2013; Li N. et al., 2022). Degenerative lumbar spondylolisthesis is predominantly observed in the adult population, exhibiting a prevalence ranging from approximately 3.2%–25% (Wang et al., 2017). Lumbar spondylolisthesis typically presents as an asymptomatic condition; however, patients frequently report symptoms such as pain in the lumbosacral region and lower extremities, paresthesia, postural alterations, and neurological deficits as the disease progresses.

Spinal stenosis (SS), a prevalent spinal degenerative condition, is typified by the narrowing of the lumbar spinal canal. The primary manifestations involve LBP and lower extremity pain, coupled with challenges in ambulation, constraints in functional capacity, and limitations in daily activity participation. These factors subsequently contribute to pronounced adverse psychological impacts (Ammendolia et al., 2017).

The gut microbiota (GM), a complex and diverse microbial community residing within the gastrointestinal tract, exists in a symbiotic relationship with its host. This intricate ecosystem is predominantly bacterial but also includes viruses, archaea, fungi, and unicellular eukaryotes (Geng et al., 2023). The intestinal microbiota is predominantly composed of approximately 1,200 species, mainly belonging to the phyla Bacteroidetes, Firmicutes, Actinobacteria, Proteobacteria, and Verrucomicrobia (Jandhyala et al., 2015). The GM, acquired at birth and predominantly maternal in origin, evolves dynamically under the influence of environmental determinants such as diet, disease, and pharmacological interventions (Plata et al., 2019). It plays a pivotal role in modulating host physiology by regulating a spectrum of processes, including inflammation, oxidative stress, immune function, and metabolic balance. Disruptions in these roles can contribute to inflammation, pain, and disease development (Jandhyala et al., 2015).

The intricate interactions between GM and musculoskeletal diseases have attracted considerable attention in recent years. Researchers have proposed and substantiated the existence of various axes, including the gut-bone axis, gut-joint axis (Qaiyum et al., 2021; Zaiss et al., 2021), gut-disk axis (Li W. et al., 2022), and gut-spine axis (Morimoto et al., 2023). Studies also reported a relationship between GM and AIS (Shen et al., 2019). Shen et al. observed significant disparities in the composition of the gut microbiota between patients with AIS and a control group consisting of healthy individuals, and a positive correlation has been identified between the abundance of fecal bacteria and the Cobb angle in AIS patients (Shen et al., 2019). However, direct evidences regarding the impact of the GM on the development of AIS, LS, and SS is scarce. The causal relationships between GM and the three conditions remain unclear.

Mendelian randomization (MR) is an innovative analytical method that provides unbiased estimates of causal relationships between phenotypes (Smith and Ebrahim, 2003; Burgess and Thompson, 2017). As a genetic epidemiological technique, MR utilizes single nucleotide polymorphisms (SNPs) highly correlated with exposures as instrumental variables (IVs) to infer the causal effects of exposures on outcomes. This approach circumvents the confounding effects of traditional factors, offering robust evidence for comprehending disease mechanisms and evolution (Davey Smith and Hemani, 2014). We aimed to employ large-scale genome-wide association study (GWAS) data for MR analysis to investigate the causal relationships between the gut microbiota and three SDDs. This approach can help to confirm existing evidence and offer fresh perspectives on the management and prevention of AIS, LS, and SS.

## 2 Materials and methods

### 2.1 Study design

**FIGURE 1:**
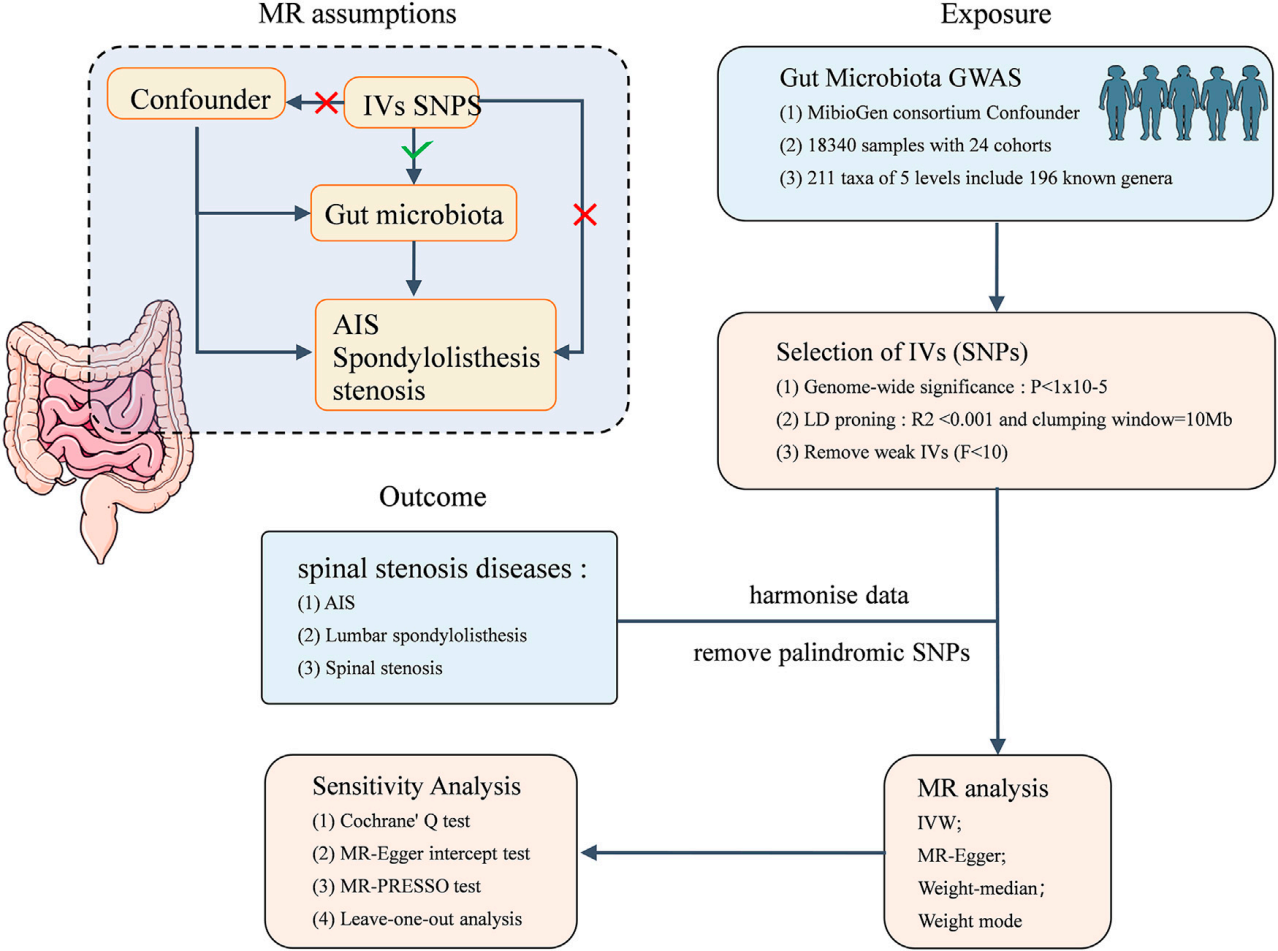
Flowchart of this MR study, and the three assumptions of MR analysis.

This study is based on summary data from a GWAS on GM and AIS, LS, and SS. We selected eligible IVs for MR studies to evaluate the causal relationship between the GM and the three musculoskeletal conditions. As shown in Figure 1, the flowchart of this study rigorously adheres to the three assumptions of MR studies: (1) the selected IVs are strongly correlated with the exposure, (2) IVs are unrelated to any confounding factors, and (3) IVs can influence the outcome only through the exposure. The datasets utilized in this study have been made publicly accessible. Ethical approval and written informed consent were obtained during the original investigation.

### 2.2 Data source for exposure

The summary data for the GM GWAS are sourced from the MiBioGen Consortium (www.mibiogen.org) (Wang J. et al., 2018). As reported by Kurilshikov et al. (2021), the MiBioGen Consortium conducted an analysis of host genotypes and gene sequencing spectra of fecal microbiota 16S rRNA from a total of 18,340 participants across 24 cohorts in various countries, including the United States, Canada, Israel, South Korea, Germany, Denmark, the Netherlands, Belgium, Sweden, Finland, and the United Kingdom. The objective of this analysis was to assess the relationships between genetic variations and the GM. Microbiome trait loci (mbTLs) were identified using standardized methods to determine genetic loci influencing the relative abundance (mbQTLs) or presence (mbBTLs) of specific microbial taxa. Finally, the biological interpretation of the GWAS results was conducted using Gene Set Enrichment Analysis (GSEA), Phenome-wide Association Studies (PheWAS) (Kurilshikov et al., 2021). The GWAS ultimately identified 122,110 variant loci across 211 taxonomic groups ranging from the phylum to the genus level.

### 2.3 Data sources for outcomes

The summary data for GWASs on AIS, LS, and SS were exclusively sourced from the FinnGen Consortium. The FinnGen research project integrates disease genetic data from the Finnish Biobank and the Finnish Health Registry with the primary objective of elucidating genotype‒phenotype correlations within the Finnish population. The GWAS for AIS included 165,850 individuals of European descent, with 1,168 cases and 164,682 controls, totaling 9 SNPs. The GWAS for LS encompasses 167,351 individuals of European descent, with 2,669 cases and 164,682 controls, involving 16,380,280 SNPs. The GWAS for SS covers 173,851 individuals of European descent, including 9,169 cases and 164,682 controls, with a dataset of 16,380,277 SNPs.

### 2.4 IVs selection

In this MR study, SNPs that are closely associated with each GM taxon are utilized as IVs. To ensure the robustness of the data and the accuracy of the results, the following selection criteria were applied for IVs: (1) SNPs associated with the GM must meet the genome-wide significance threshold (P < 5 × 10^−8^). Given the limited number of qualifying IVs available, a relatively comprehensive threshold was chosen (P < 1 × 10^−5^) (Geng et al., 2023). (2) To guarantee the independence of each IV, linkage disequilibrium (LD) analysis was conducted (R2 < 0.001, clumping distance = 10,000 kb), and SNPs not meeting the criteria were removed. (3) Palindromic SNPs are excluded to mitigate the potential influence of allelic genes on the inferred causal relationships among GM taxa, AIS, LS, and SS.

### 2.5 Statistical analysis

Four different methods, including Inverse Variance Weighting (IVW), MR Egger, Weighted Median, and Weighted Mode, were employed in the MR analysis to examine the causal relationship between the GM and the conditions of AIS, LS, and SS, utilizing a set of selected IVs. Among these, the IVW approach was primarily utilized for determining significant causal associations (P < 0.05), while the remaining methods served as supplementary measures. The IVW method employs a meta-analysis approach, conducting Wald ratio assessments for the causal relationships generated by various SNPs to ensure a robust evaluation of the causal association between the exposure and outcom (Li P. et al., 2022). MR‒Egger regression is predicated on the assumption of instrument strength that is independent of direct effect (InSIDE), thereby facilitating the evaluation of horizontal pleiotropy through the intercept term. In case the intercept term equals 0, it indicates the absence of horizontal pleiotropy, and the results of MR‒Egger results are consistent with the IVW results. Even in scenarios where up to 50% of IVs are proven ineffective, the weighted median method remains capable of accurately estimating the causal relationship between exposure and outcome (Bowden et al., 2016). If the assumption of InSIDE is violated, the weighted mode model demonstrates reduced bias and a lower Type I error when assessing causal relationships compared to MR‒Egger (Hartwig et al., 2017).

The Cochran’s Q statistic was used to assess heterogeneity among SNPs, and when P > 0.05 indicated a low potential for heterogeneity, the analysis utilized the IVW fixed-effects model. Conversely, the IVW random-effects model was applied when P ≤ 0.05 (Pagoni et al., 2019). Horizontal pleiotropy was examined using the MR‒Egger intercept test, where P > 0.05 indicated an absence of horizontal pleiotropy. Additionally, the MR-PRESSO global test was conducted to detect and eliminate outliers, thereby mitigating the impact of pleiotropy (Verbanck et al., 2018). Furthermore, a “Leave One Out” analysis was employed to investigate whether the causal relationship between the gut microbiota and the aforementioned three diseases is influenced by individual SNPs.

All the statistical analyses were conducted using R version 4.2.1. The TwoSampleMR package (version 0.5.7) and MR-PRESSO package (version 1.0) in R were used for the MR analyses.

## 3 Results

### 3.1 Selection of IVs related to the GM

After excluding 3 unknown families and 12 unknown genera from the initial set of 211 gut microbiota, a total of 196 microbial taxa were included as exposures. According to the IVs selection criteria, 2035 SNPs associated with 193 bacterial taxa were identified as instrumental variables. These classifications comprised 9 phyla (103 SNPs), 16 classes (179 SNPs), 20 orders (217 SNPs), 31 families (339 SNPs), and 117 genera (1,197 SNPs). Detailed information regarding the selected instrumental variables is provided in the Supplementary File S1.

### 3.2 Causal effects of the gut microbiota on AIS

Individual MR analyses were conducted for each of the three diseases. Causal Associations were identified between 9 GM taxa (9 Genera) and AIS. As shown in Table 1, the IVW analysis revealed that genus Eubacteriumventriosum (OR = 1.692, 95%CI: 1.142–2.508, P = 0.009), genus Catenibacterium (OR = 1.596, 95%CI: 1.108–2.298, P = 0.012), genus LachnospiraceaeFCS020 (OR = 1.592, 95%CI: 1.068–2.371, P = 0.022), genus Ruminiclostridium6 (OR = 1.514, 95%CI: 1.025–2.239, P = 0.037), genus RuminococcaceaeUCG009 (OR = 1.393, 95%CI: 1.024–1.895, P = 0.035), and genus Desulfovibrio (OR = 1.521, 95%CI: 1.019–2.272, P = 0.040) were associated with an increased risk of AIS. Conversely, genus Eubacterium eligens (OR = 0.468, 95%CI: 0.231–0.951, P = 0.036), Bilophila (OR = 0.609, 95%CI: 0.374–0.991, P = 0.046), and Prevotella9 (OR = 0.687, 95%CI: 0.511–0.924, P = 0.013) were associated with a reduced risk of AIS.

**TABLE 1:** MR estimates for the association between GM and AIS.

Group	Gut microbiota	Methods	Nsnp	OR	OR (95%CI)	p-value
Genus	Eubacteriumventriosumgroup	IVW	15	1.692	1.142–2.508	0.009
		MR Egger		1.089	0.189–6.285	0.925
		Weighted median		1.846	1.076–3.169	0.026
		Weighted mode		2.787	0.978–7.948	0.076
Genus	Catenibacterium	IVW	4	1.596	1.108–2.298	0.012
		MR Egger		0.810	0.008–78.263	0.936
		Weighted median		1.542	0.982–2.423	0.060
		Weighted mode		1.408	0.799–2.481	0.322
Genus	LachnospiraceaeFCS020group	IVW	12	1.592	1.068–2.371	0.022
		MR Egger		1.814	0.627–5.252	0.298
		Weighted median		1.740	1.013–2.990	0.045
		Weighted mode		2.478	1.006–6.106	0.074
Genus	Ruminiclostridium6	IVW	14	1.514	1.025–2.239	0.037
		MR Egger		2.119	0.824–5.452	0.145
		Weighted median		1.381	0.817–2.335	0.228
		Weighted mode		1.303	0.560–3.029	0.549
Genus	RuminococcaceaeUCG009	IVW	12	1.393	1.024–1.895	0.035
		MR Egger		0.978	0.275–3.476	0.974
		Weighted median		1.660	1.097–2.514	0.017
		Weighted mode		1.946	0.931–4.067	0.105
Genus	Desulfovibrio	IVW	9	1.521	1.019–2.272	0.040
		MR Egger		1.772	0.542–5.791	0.375
		Weighted median		1.490	0.898–2.474	0.123
		Weighted mode		1.470	0.666–3.245	0.369
Genus	Eubacteriumeligensgroup	IVW	6	0.468	0.231–0.951	0.036
		MR Egger		0.125	0.009–1.753	0.198
		Weighted median		0.475	0.207–1.089	0.079
		Weighted mode		0.490	0.160–1.498	0.266
Genus	Bilophila	IVW	13	0.609	0.374–0.991	0.046
		MR Egger		0.334	0.028–3.965	0.403
		Weighted median		0.547	0.301–0.992	0.047
		Weighted mode		0.527	0.157–1.767	0.320
Genus	Prevotella9	IVW	15	0.687	0.511–0.924	0.013
		MR Egger		0.468	0.197–1.112	0.109
		Weighted median		0.687	0.455–1.037	0.074
		Weighted mode		0.703	0.372–1.328	0.296

### 3.3 Causal effects of the gut microbiota on lumbar spondylolisthesis

Causal associations were identified between 14 GM taxa (4 phyla, 2 classes, 2 orders, 1 family, and 5 genera) and LS. As shown in Table 2, the IVW analysis revealed the following: phylum Actinobacteria (OR = 1.439, 95%CI: 1.078–1.921, P = 0.014), phylum Proteobacteria (OR = 1.582, 95%CI: 1.039–2.409, P = 0.033), class Coriobacteria (OR = 1.414, 95%CI: 1.040–1.922, P = 0.027), order Coriobacteriales (OR = 1.414, 95%CI: 1.040–1.922, P = 0.027), family Coriobacteriaceae (OR = 1.414, 95%CI: 1.040–1.922, P = 0.027), genus Parabacteroides (OR = 1.723, 95%CI: 1.020–2.910, *p* = 0.042), genus RuminococcaceaeNK4A214group (OR = 1.435, 95%CI: 1.045–1.971, P = 0.026), genus Sutterella (OR = 1.404, 95%CI: 1.038–1.899, P = 0.028). On the other hand, phylum Firmicutes (OR = 0.751, 95%CI: 0.578–0.976, P = 0.032), phylum Lentisphaerae (OR = 0.812, 95%CI: 0.670–0.983, P = 0.033), class Lentisphaeria (OR = 0.778, 95%CI: 0.633–0.955, P = 0.016), order Victivallales (OR = 0.778, 95%CI: 0.633–0.955, P = 0.016), genus Romboutsia (OR = 0.747, 95%CI: 0.563–0.990, P = 0.042), and genus Senegalimassilia (OR = 0.671, 95%CI: 0.478–0.941, P = 0.021) were associated with a reduced risk of spondylolisthesis.

**TABLE 2:** MR estimates for the association between the GM and lumbar spondylolisthesis.

Group	Gut microbiota	Methods	Nsnp	OR	OR (95%CI)	p-value
Phylum	Actinobacteria	IVW	14	1.439	1.078–1.921	0.014
		MR Egger		0.510	0.154–1.696	0.294
		Weighted median		1.312	0.894–1.926	0.166
		Weighted mode		1.288	0.777–2.133	0.344
Phylum	Proteobacteria	IVW	12	1.582	1.039–2.409	0.033
		MR Egger		3.330	1.087–10.205	0.061
		Weighted median		1.537	1.011–2.338	0.045
		Weighted mode		1.472	0.856–2.531	0.190
Phylum	Firmicutes	IVW	15	0.751	0.578–0.976	0.032
		MR Egger		0.604	0.327–1.116	0.132
		Weighted median		0.717	0.502–1.023	0.067
		Weighted mode		0.649	0.376–1.119	0.142
Phylum	Lentisphaerae	IVW	9	0.812	0.670–0.983	0.033
		MR Egger		1.118	0.539–2.318	0.774
		Weighted median		0.790	0.621–1.004	0.054
		Weighted mode		0.754	0.517–1.099	0.180
Class	Coriobacteriia	IVW	13	1.414	1.040–1.922	0.027
		MR Egger		1.223	0.326–4.587	0.771
		Weighted median		1.466	0.971–2.215	0.069
		Weighted mode		1.382	0.656–2.909	0.411
Class	Lentisphaeria	IVW	8	0.778	0.633–0.955	0.016
		MR Egger		1.039	0.502–2.150	0.922
		Weighted median		0.782	0.603–1.015	0.065
		Weighted mode		0.754	0.500–1.137	0.220
Order	Coriobacteriales	IVW	13	1.414	1.040–1.922	0.027
		MR Egger		1.223	0.326–4.587	0.771
		Weighted median		1.466	0.942–2.282	0.090
		Weighted mode		1.382	0.687–2.780	0.382
Order	Victivallales	IVW	8	0.778	0.633–0.955	0.016
		MR Egger		1.039	0.502–2.150	0.922
		Weighted median		0.782	0.601–1.019	0.069
		Weighted mode		0.754	0.510–1.115	0.200
Family	Coriobacteriaceae	IVW	13	1.414	1.040–1.922	0.027
		MR Egger		1.223	0.326–4.587	0.771
		Weighted median		1.466	0.952–2.259	0.083
		Weighted mode		1.382	0.684–2.790	0.385
Genus	Parabacteroides	IVW	5	1.723	1.020–2.910	0.042
		MR Egger		13.935	0.772–251.578	0.172
		Weighted median		1.783	0.952–3.337	0.071
		Weighted mode		1.792	0.727–4.415	0.273
Genus	RuminococcaceaeNK4A214group	IVW	13	1.435	1.045–1.971	0.026
		MR Egger		1.349	0.453–4.020	0.601
		Weighted median		1.056	0.706–1.578	0.791
		Weighted mode		0.955	0.522–1.747	0.884
Genus	Sutterella	IVW	12	1.404	1.038–1.899	0.028
		MR Egger		1.369	0.347–5.405	0.663
		Weighted median		1.521	1.034–2.236	0.033
		Weighted mode		1.575	0.891–2.785	0.146
Genus	Romboutsia	IVW	13	0.747	0.563–0.990	0.042
		MR Egger		0.767	0.325–1.809	0.557
		Weighted median		0.698	0.473–1.030	0.070
		Weighted mode		0.678	0.343–1.342	0.286
Genus	Senegalimassilia	IVW	5	0.671	0.478–0.941	0.021
		MR Egger		0.696	0.188–2.573	0.625
		Weighted median		0.714	0.465–1.098	0.125
		Weighted mode		0.724	0.405–1.292	0.336

### 3.4 Causal effects of the gut microbiota on spinal stenosis

Causal associations were identified between 4 GM taxa (2 classes, 1 order, and 1 genus) and SS. As shown in Table 3, the IVW analysis revealed that class Clostridia (OR = 1.196, 95%CI: 1.004–1.424, P = 0.044), class Gammaproteobacteria (OR = 1.374, 95%CI: 1.062–1.779, P = 0.016), order Clostridiales (OR = 1.196, 95%CI: 1.008–1.420, P = 0.040), and genus Oxalobacte (OR = 1.132, 95%CI: 1.028–1.247, P = 0.011) were associated with an increased risk of stenosis.

**TABLE 3:** MR estimates for the association between the GM and spinal stenosis.

Group	Gut microbiota	Methods	Nsnp	OR	OR (95%CI)	p-value
Class	Clostridia	IVW	12	1.196	1.004–1.424	0.044
		MR Egger		0.933	0.581–1.500	0.782
		Weighted median		1.340	1.050–1.710	0.019
		Weighted mode		1.488	1.021–2.167	0.063
Class	Gammaproteobacteria	IVW	6	1.374	1.062–1.779	0.016
		MR Egger		1.692	0.774–3.700	0.258
		Weighted median		1.348	0.984–1.847	0.063
		Weighted mode		1.358	0.878–2.101	0.227
Order	Clostridiales	IVW	13	1.196	1.008–1.420	0.040
		MR Egger		0.997	0.625–1.590	0.990
		Weighted median		1.144	0.895–1.463	0.281
		Weighted mode		1.292	0.914–1.823	0.172
Genus	Oxalobacte	IVW	11	1.132	1.028–1.247	0.011
		MR Egger		1.247	0.794–1.959	0.362
		Weighted median		1.164	1.028–1.319	0.017
		Weighted mode		1.176	0.962–1.437	0.146

**FIGURE 2:**
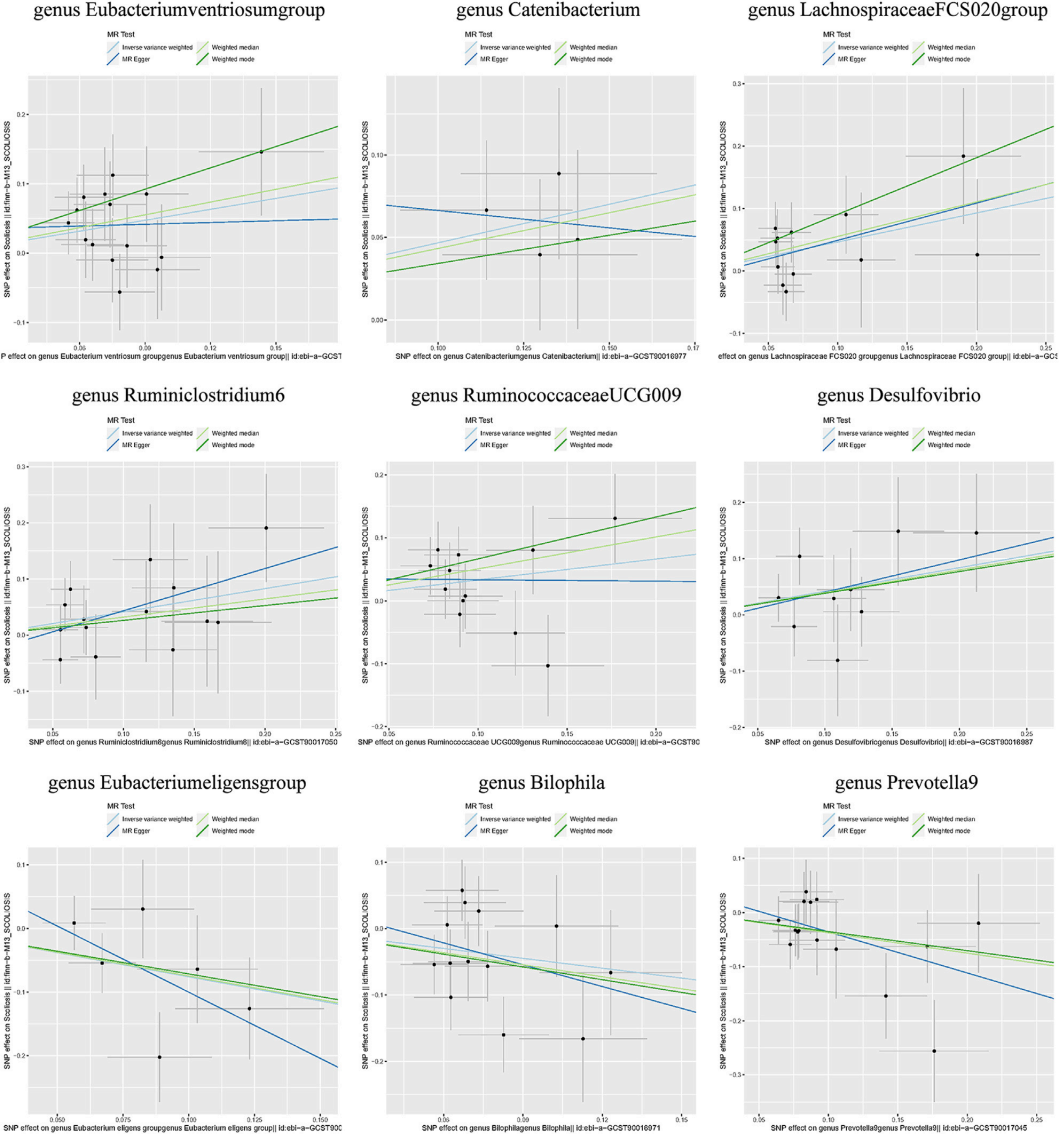
Scatter plots for the causal association between the GM and AIS.

**FIGURE 3:**
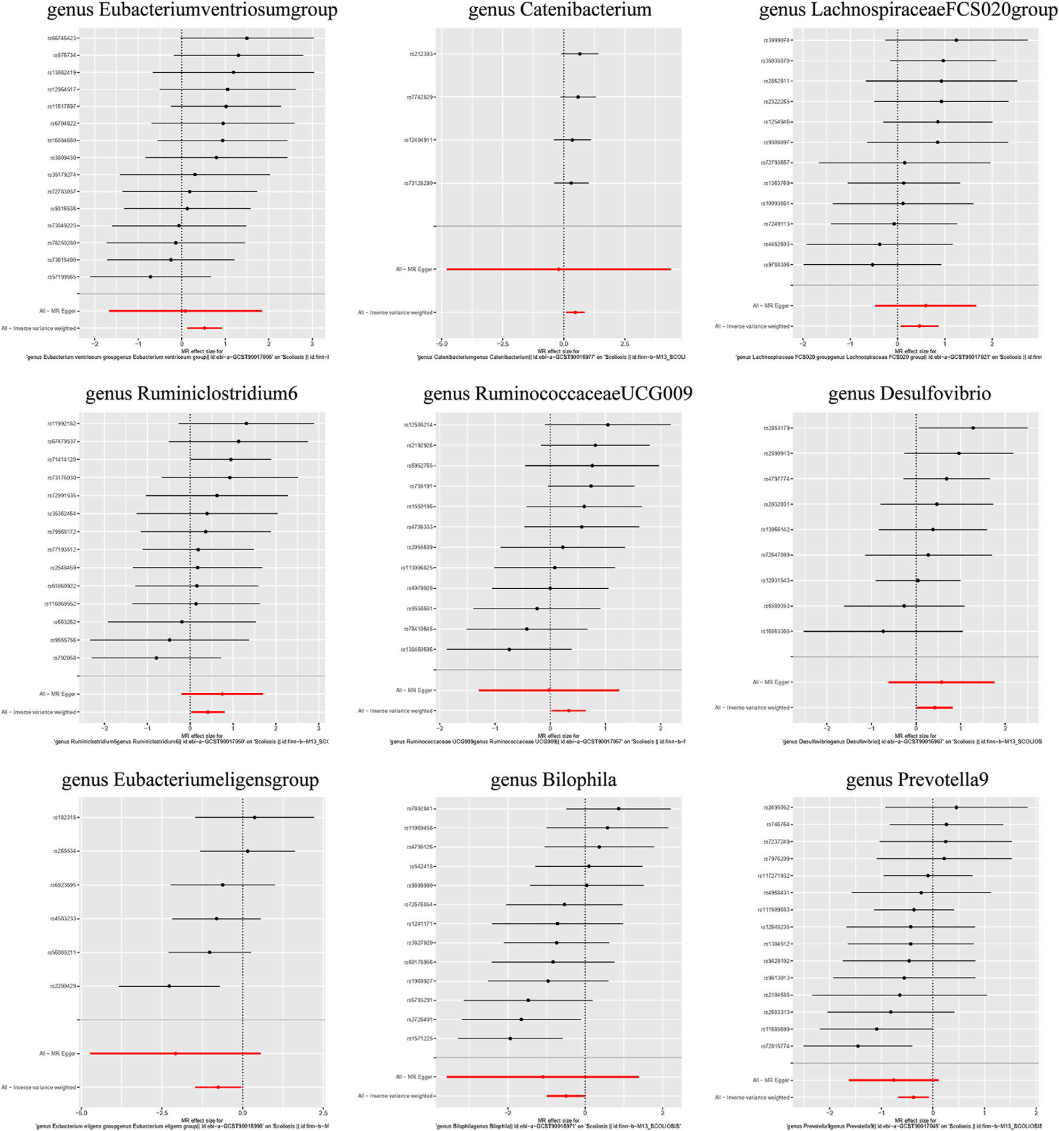
Forest plots for the causal association between the GM and AIS.

**FIGURE 4:**
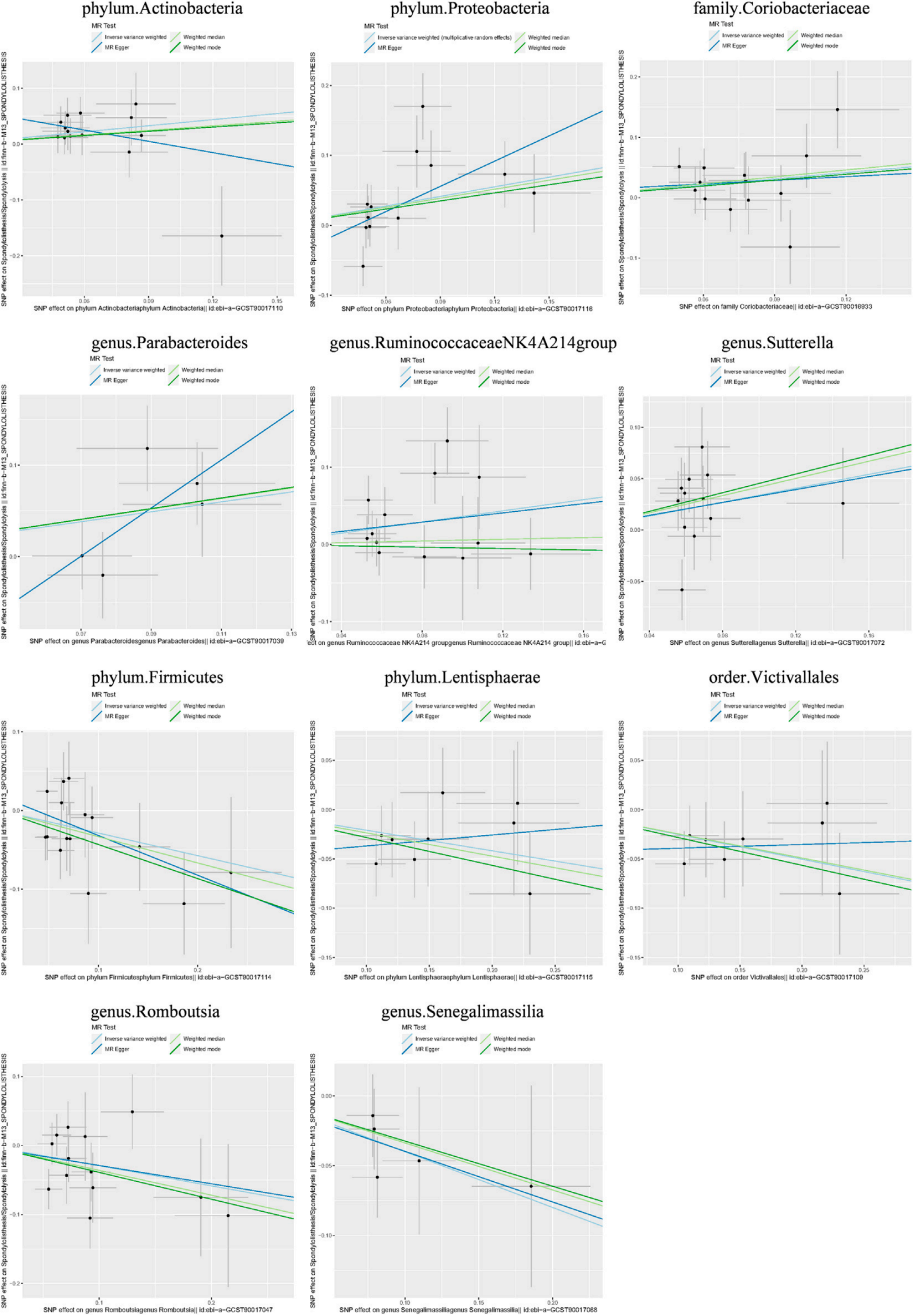
Scatter plots for the causal association between the GM and lumbar spondylolisthesis.

**FIGURE 5:**
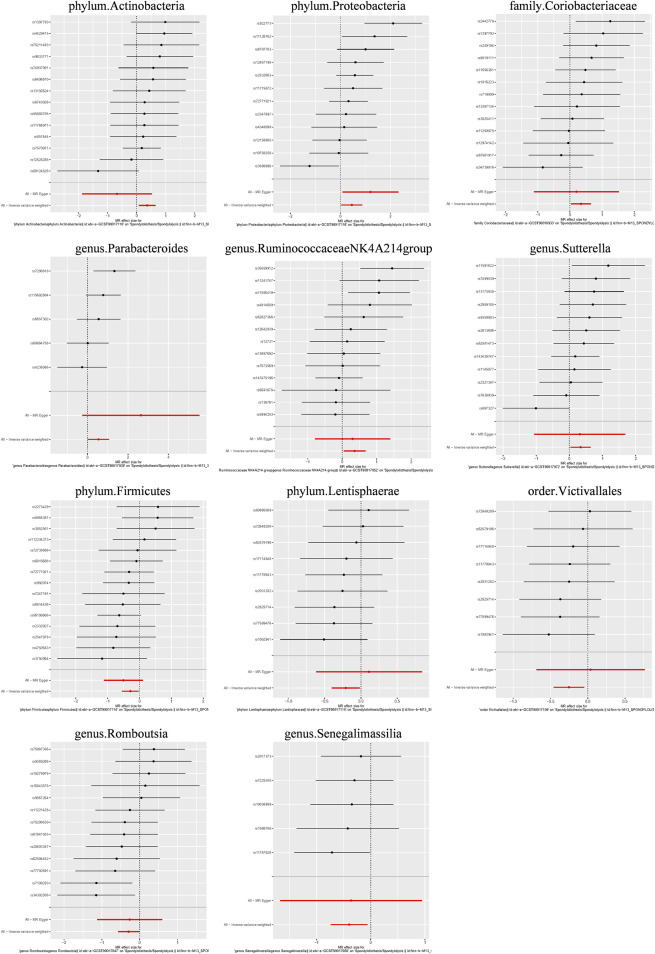
Forest plots for the causal association between the GM and lumbar spondylolisthesis.

**FIGURE 6:**
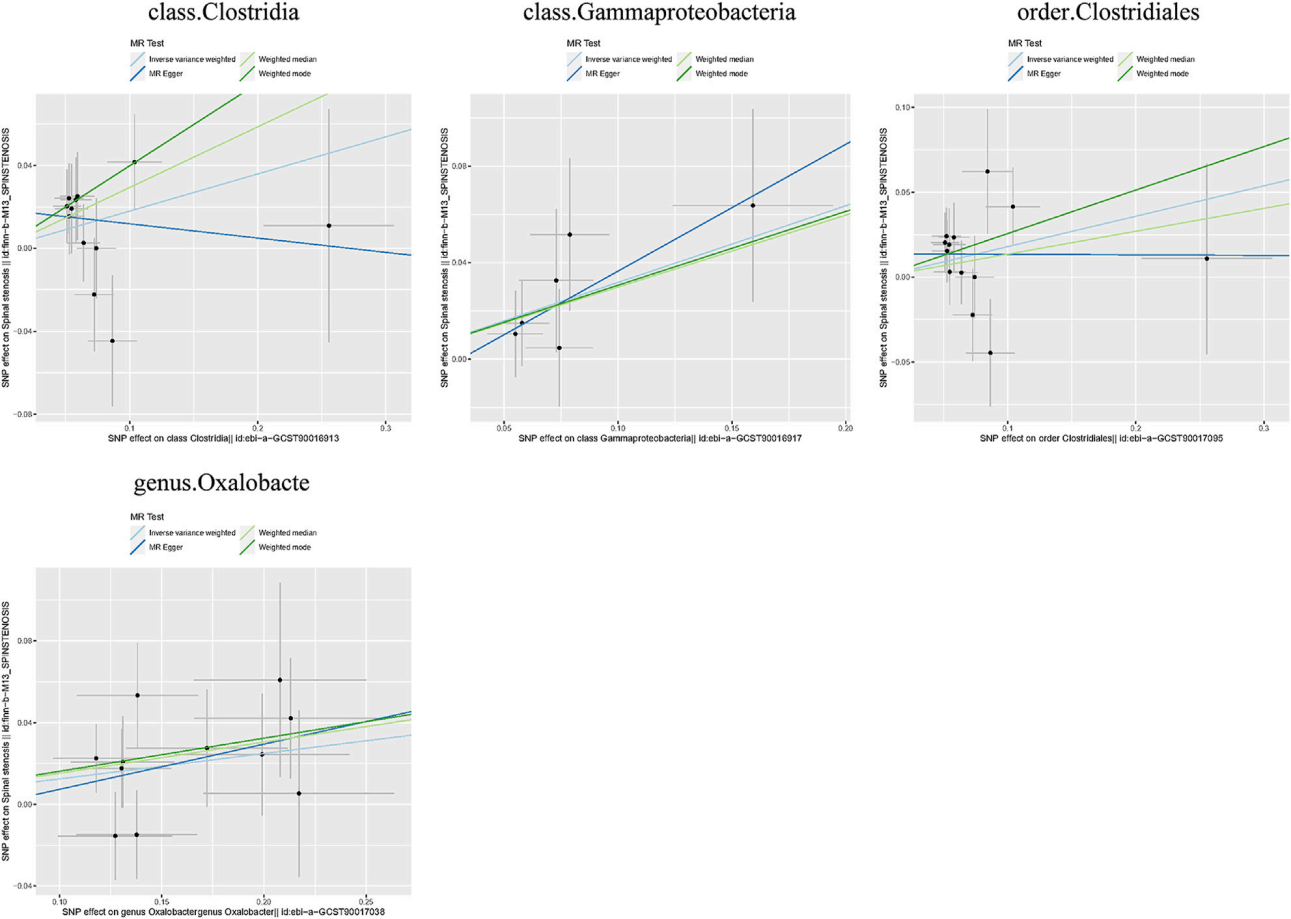
Scatter plots for the causal association between the GM and spinal stenosis.

**FIGURE 7:**
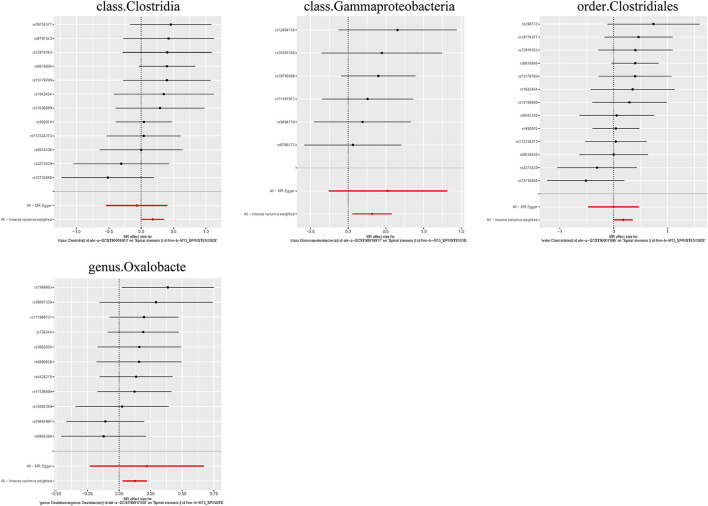
Forest plots for the causal association between the GM and spinal stenosis.

**FIGURE 8:**
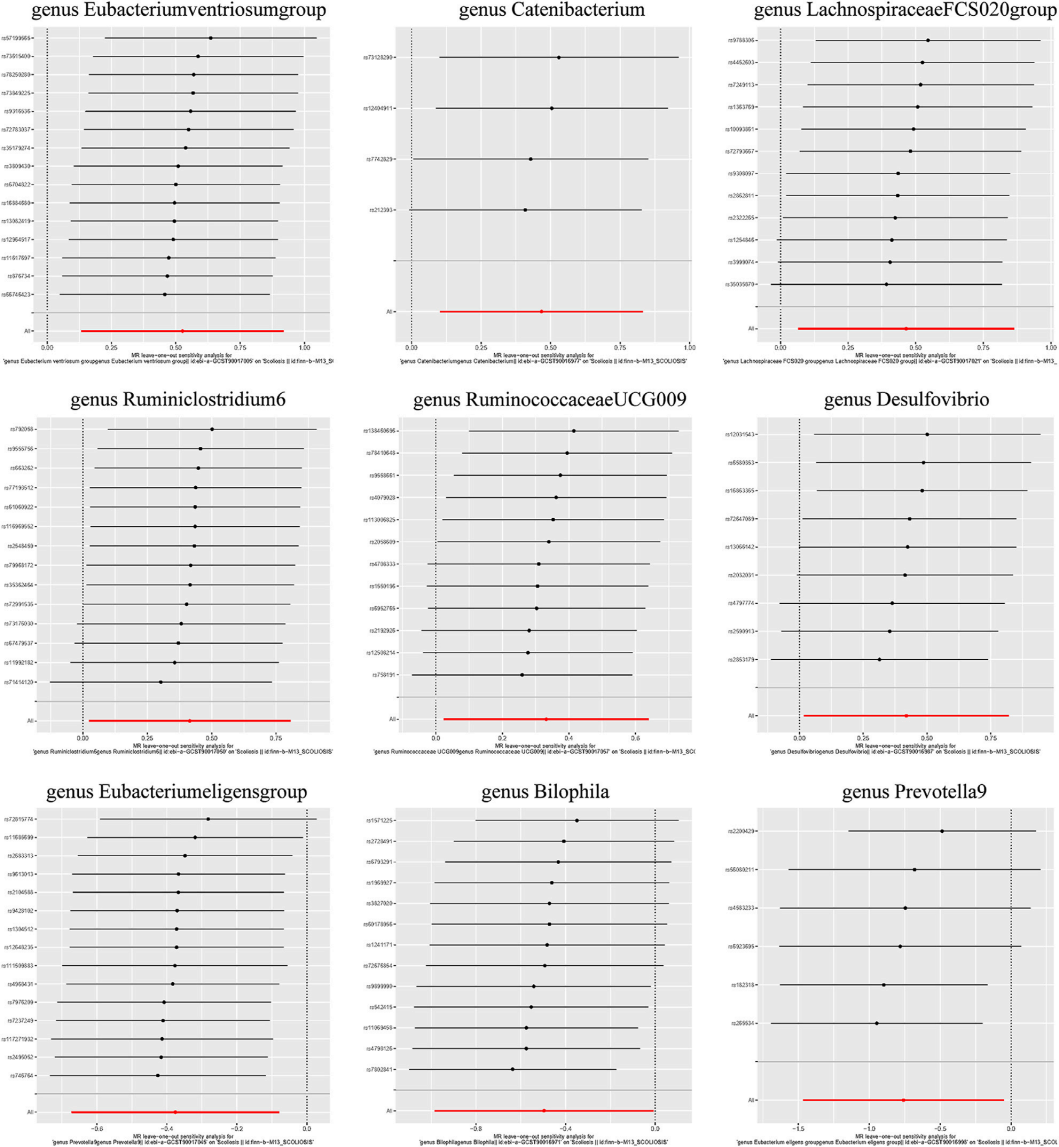
Leave-one-out plots for the causal association between the GM and AIS.

**FIGURE 9:**
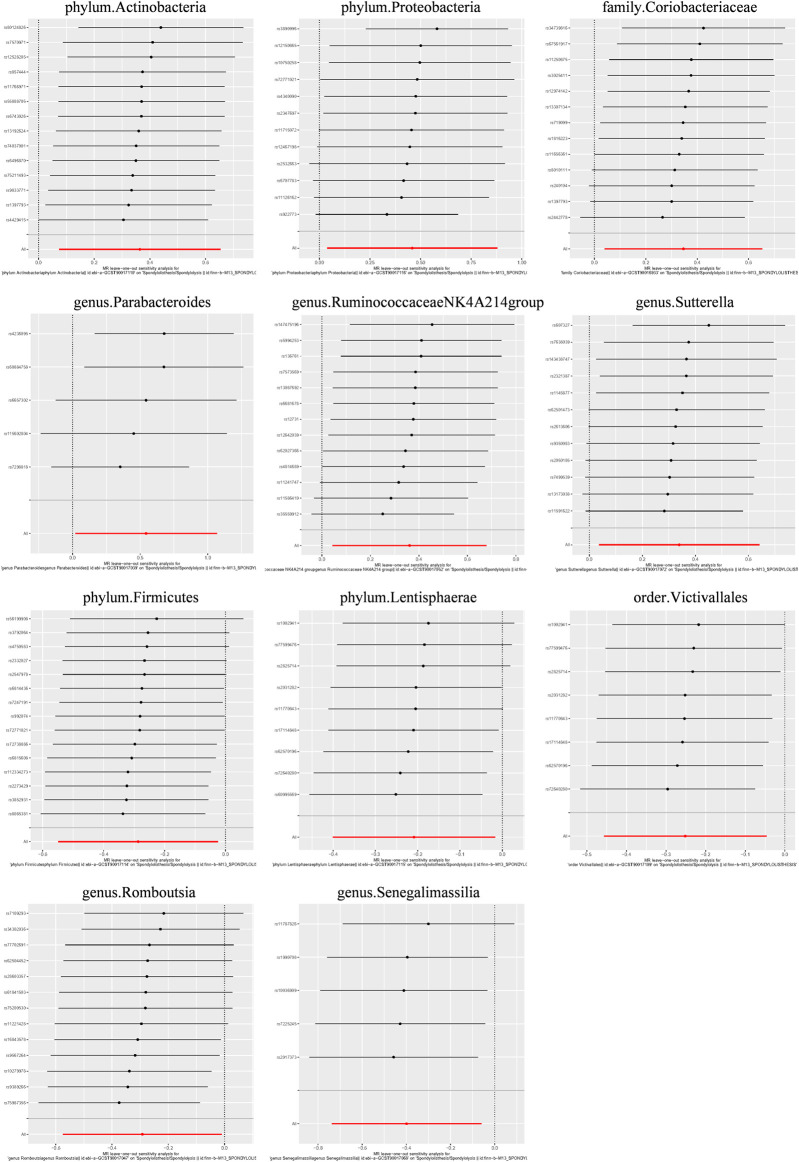
Leave-one-out plots for the causal association between the GM and lumbar spondylolisthesis.

**FIGURE 10:**
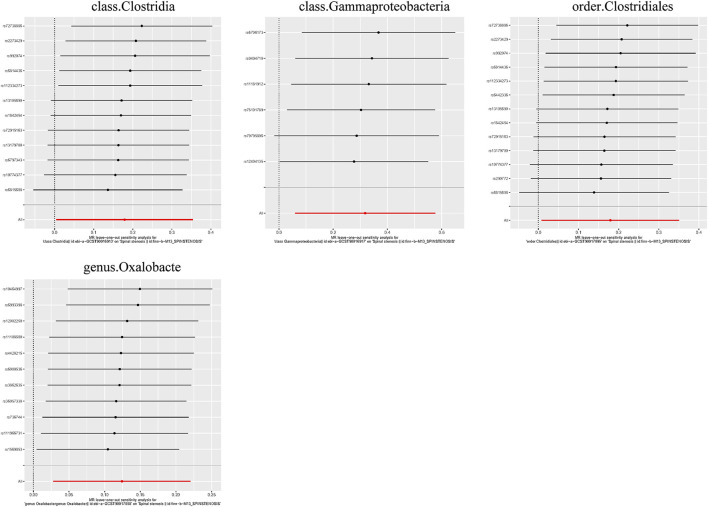
Leave-one-out plots for the causal association between the GM and spinal stenosis.

Additionally, three supplementary methods were employed for further analysis, and the results were consistent with the direction of the IVW method, indicating the robustness of the IVW results. Scatter plots and forest plots indicated that there were no potential outliers that could significantly affect the estimates (Figures 2–7). The leave-one-out results showed that no SNPs with strong effects significantly affected the outcome estimates (Figures 8–10).

## 4 Discussion

Spinal stenosis disease is caused by central or neuroforaminal narrowing from lumbar spinal anatomical derangements that can be congenital or acquired. Symptoms of SSD result from direct compression or ischemia of the neural elements. Patients may present with symptoms such as neurogenic claudication, radicular pain, low back pain, and cauda equina syndrome. Compression of the lateral recesses or the central cord may lead to lower urinary tract symptoms, which is marked by impairment in intraneuronal blood flow, intraneuronal oedema, structural damage to the neurons and axonal transport block (Gandhi et al., 2018; Lucke-Wold et al., 2020).

For most individuals, initial treatment should focus on education, medications to control pain, exercise and physical treatments to regain or maintain activities of daily living. In the older patient population, multidisciplinary treatments such as strength and endurance training, flexibility exercises, lifestyle modification, and environment modifications have shown positive results. However, there is insufficient evidence to support the use of physical therapy/exercise or manipulation treatment for spinal stenosis (Deer et al., 2019). Surgical treatment most commonly is considered in patients not improving with nonsurgical care. Decompressive laminectomy is the standard surgical procedure for patients with spinal stenosis. The addition of fusion with or without instrumentation is considered when spinal stenosis is accompanied by degenerative spondylolisthesis or related to concerns about instability.

A stable ecology of gut microbiota enhances intestinal permeability, mitigates inflammation, and actively engages in immune regulation. However, the onset of GM dysbiosis can precipitate the selective enrichment of certain microorganisms, leading to the production of microbial derivatives or metabolites that contribute to the development of various systemic diseases (Kho and Lal, 2018). GM releases short-chain fatty acids (SCFAs) that regulate G-protein-coupled receptors, influencing hormone secretion, neurotransmitter release and modulating inflammation and mood. Furthermore, unfavorable alterations in the composition of the microbiota may be associated with an increased risk of neurological injuries due to elevated levels of proinflammatory molecules and clotting factors. It is noteworthy that neurological injuries, such as ischemic stroke and spinal cord injury have been shown to lead to gut dysbiosis (Panther et al., 2022). This may help to explain why SSD patients frequently suffer from symptoms such as neurogenic claudication, radicular pain, and low back pain.

Accumulating data support the significant role of GM in musculoskeletal disorders and its involvement in the onset and progression of related diseases. Substantial researches have implicated the composition of GM in the pathogenesis of musculoskeletal conditions including rheumatoid arthritis, ankylosing spondylitis (Asquith et al., 2019), osteoarthritis (Biver et al., 2019), spondylitis (Breban et al., 2017), intervertebral disc degeneration (Li W. et al., 2022), and osteoporosis (Rajasekaran et al., 2020), through various pathways. GM dysbiosis exert detrimental effects on spinal structural health through 3 primary mechanisms: (1) perturbations in nutritional pathways, including those of calcium, amino acids, and vitamin K, which are essential for bone metabolism; (2) immune modulation associated with estrogen levels, SCFA, and systemic inflammatory responses, which can alter bone homeostasis; and (3) the influence of neurotransmitters such as serotonin and leptin, which have been implicated in the regulation of bone metabolism, leading to a disruption in the balance between osteoblast and osteoclast activity (Chen et al., 2022).

Vitamin D, known for its role in calcium metabolism, has been hypothesized by Ng et al. to also influence fibrosis regulation and postural control, which could be instrumental in the development of AIS (Ng et al., 2018). A study on the impact of vitamin D on the gut-bone axis demonstrated that mice with deficient vitamin D levels exhibited a diminished abundance of *Bacteroides*/Prevotella, and protracted vitamin D deficiency is associated with the potential to induce inflammation (Jahani et al., 2014). Although direct evidence is limited, it is plausible that a decrease in Prevotella abundance could impact vitamin D levels, initiate inflammatory responses, and subsequently contribute to the development of AIS. However, some findings contradict the results of our study. Shen et al., in their investigation of the intestinal microbiota of AIS patients, reported a significant increase in Prevotella abundance, which showed a positive correlation with the Cobb angle. They propose that Prevotella might be involved in the pathogenesis of AIS through inflammatory pathways (Shen et al., 2019). The degree of curvature of the AIS has been correlated with decreased bone mineral density, possibly due to the activation of osteoclasts and the suppression of osteoblast expression (Zhou et al., 2012), and increased prevalence of Prevotella has been strongly linked with reduced bone density (Jiminez et al., 2016). However, there is insufficient foundational research to conclusively support the direct impact of Prevotella on the onset and progression of AIS.

Toddalia asiatica extract (TAE) has been demonstrated that it can ameliorate rheumatoid arthritis (RA) symptoms, modulate the intestinal microbiota, and reduce the abundance of harmful bacteria linked to RA, including those of the Eubacterium eligens group. Additionally, TAE increased the abundance of beneficial bacteria for RA, such as Lachnospiraceae and Ruminococcaceae. Gao et al. proposed that the dysregulation of CD4^+^ T-cell subgroups contribute to the pathogenesis of RA. TAE may alleviate RA symptoms by modulating the expression of Th17/Treg cells, while the Eubacterium eligens group is implicated in the inflammatory pathway associated with TAE (Gao et al., 2022). Further research is necessary to elucidate the specific pathways through which these microbial factors influence the development and progression of AIS.

As core members of the human intestinal flora, genus Parabacteroides, genus Eubacterium ventriosum group, phylum Firmicutes, and family Ruminococcaceae all can produce SCFAs, including acetate, propionate, and primarily butyrate (Liu et al., 2019; Ren et al., 2019). Butyric acid serves as a primary energy source for colonic cells and plays a vital role in maintaining intestinal barrier integrity while mitigating inflammation (Wei et al., 2018). As metabolites derived from the gut microbiota, SCFAs exhibit host anti-inflammatory effects, regulate gut microbiota homeostasis, and provide energy for intestinal epithelial function. Consequently, a decrease in the populations of these SCFA-producing bacteria can contribute to inflammation, ultimately leading to the development of SSD.

The genus Bilophila can produce hydrogen sulfide, which serves as a physiological modulator by inducing smooth muscle relaxation in the ileum and promoting increased blood supply to the gastrointestinal mucosa (Zhao et al., 2019). Yang et al. have reported an association between elevated hydrogen sulfide production and increased levels of Bilophila in postmenopausal women, which may precipitate local inflammation and mucosal damage (Yang et al., 2022). However, our study’s findings appear to contradict the established pro-inflammatory pathways. It is plausible that Bilophila could mitigate the onset and progression of AIS through mechanisms that are distinct from its effects on inflammatory processes.

The pathophysiological process underlying lumbar spondylolisthesis encompasses various factors, including facet joint osteoarthritis and dysfunction of ligaments and paravertebral muscles. Inflammation may represent a pivotal pathway by which the gut microbiota is implicated in lumbar spondylolisthesis. In recent studies, the class Coricoriobacteriia has emerged as a potential biomarker in the inflammatory model of depression (Huang et al., 2019). A study on nonalcoholic steatohepatitis revealed a significant increase in the abundance of the family Coriobacteriaceae in mice (Li et al., 2021). Another study demonstrated an inverse correlation between the abundance of the class Coriobacteriia and the abundance of the order Coriobacteriales and high-density lipoprotein (HDL) levels (Guo et al., 2022). The potential influence of Coriobacteriia and Coriobacteriales on nonalcoholic fatty liver disease may be attributed to their impact on HDL levels, considering the anti-inflammatory and antioxidant properties associated with HDL (Wang Y. et al., 2018). Therefore, we suggest that the class Coriobacteriia, family Coriobacteriaceae, and order Coriobacteriales as potential risk factors that may contribute to the occurrence and progression of degenerative slip through inflammatory pathways.

Recent studies have suggested a potential link between the class Lentisphaeria and immune regulation. An increased abundance of the class Lentisphaeria has been observed in individuals diagnosed with inflammatory bowel disease, whereas its occurrence appears to be diminished among patients suffering from rosacea (Fan et al., 2020; Chen et al., 2021). Additionally, the presence of class Lentisphaeria was found to be associated with a reduction in symptom severity scores among patients diagnosed with posttraumatic stress disorder (PTSD) (Hemmings et al., 2017). Notably, previous studies have reported a functional correlation between the class Lentisphaeria and fibroblast activation (Zhan et al., 2021). Fibrosis of the ligaments surrounding the lumbar spine and paravertebral muscles in chronic inflammatory responses leads to compromised support and protective functions, potentially serving as a fundamental factor contributing to lumbar spondylolisthesis. Therefore, further investigation into the role of class Lentisphaeria in modulating the development or progression of lumbar spondylolisthesis is warranted.

The immune response is regulated by a diverse range of mediators, including chemokines and cytokines, which play crucial roles in both bone resorption and bone formation. Studies have indicated that gastrointestinal disorders may accelerate bone loss by promoting osteoclastogenesis through the immune-mediated cytokine receptor activator of nuclear factor kappa-B ligand (RANKL) pathway, thereby affecting the activity and remodeling processes of bone cells (Moore, 2016; Daneshmand et al., 2022). *Clostridium* infection induces the upregulation of several cytokines, including TNF-α, IL-1β, IL-6 and IL-17, which play crucial roles in the expression of RANKL and the promotion of osteoclastogenesis (Alizadeh et al., 2021; Graham et al., 2023). TNF-α serves as a potent inhibitor of various transcription factors involved in osteoblast differentiation, thereby attenuating the phenotypic selection of precursor cells for the osteoblast pathway (Kotake and Nanke, 2014). Among the proinflammatory cytokines, IL-17 secreted by Th17 cells exhibits the highest level of RANKL expression, exerting a robust osteoclastic effect that is upregulated during infection. Furthermore, IL-17 amplifies local inflammation, and the production of TNF-α and IL-6 further enhances RANKL expression (Sato et al., 2006). This may explain the positive effect of *Clostridium* on spinal stenosis in this study.

Oxalobacter, a gram-negative obligate anaerobe, is recognized as a crucial constituent of the normal intestinal microbiota in humans. This bacterium exhibits a distinctive metabolic dependency on oxalic acid as its carbon source for ATP generation. Hatch et al. have demonstrated a strong inverse correlation between the colonization of Oxalobacter and the recurrence of calcium oxalate kidney stones (Hatch et al., 2006). Previous investigations have observed significant enhancements in crystal deposition within degenerated intervertebral discs, particularly those of pyrophosphate and oxalate. These crystals are known to compromise the extracellular matrix of the discs and exacerbate pre-existing degenerative alterations through the upregulation of matrix metalloproteinases (Gruber et al., 2007). Our study indicated a positive association between the presence of Oxalobacter and the prevalence of spinal stenosis, which seems at odds with existing literature that suggests Oxalobacter may diminish oxalate deposition in tissues (Ermer et al., 2023). This discrepancy underscores the need for further research to clarify the role of Oxalobacter in the pathogenesis of spinal diseases.

The study identified causal relationships between GM and SSD, suggesting that targeting specific bacterial groups could represent a novel approach for the conservative treatment of SSD. Interventions such as the administration of probiotics and prebiotics, fecal microbiota transplantation (FMT), and oral SCFAs have been shown to stabilize and enhance the composition of the GM (Panther et al., 2022). Among these, probiotics have demonstrated the most promise as adjunctive therapy for alleviating symptoms associated with various neurological diseases. The therapeutic effects of probiotics may be attributed to anti-apoptotic mechanisms, including the downregulation of Bax and caspase-3, the upregulation of Bcl-2 expression, and other anti-inflammatory pathways (Li et al., 2018). FMT is an emerging technique gaining traction in the recovery from neurological injuries. Its benefits, including improved functional and behavioral recovery in animal models, are largely attributed to increased SCFA production and the reduction of harmful metabolites such as trimethylamine N-oxide (TMAO). The potential impact of orally administered SCFAs on neurological injury outcomes is still under investigation, but they may eventually serve as a safe and convenient adjunct to post-neurological injury therapy, contributing to improved patient outcomes (Panther et al., 2022). However, the current body of human clinical studies on this topic is insufficient, and further research is needed to establish the safety and efficacy of these interventions in this patient population before clinical recommendations can be made.

To the best of our knowledge, this study represents the first application of Mendelian randomization to investigate the causal associations between the GM and spinal stenosis diseases. This approach offers a novel perspective on the mechanisms underlying the development of spinal stenosis. The primary strength of this study lies in the use of Mendelian randomization analysis, which effectively mitigates confounding factors and reverse causality, thereby enhancing the credibility of the findings compared to traditional observational studies. Gut microbiota and metabolic pathways identified as having causal relationships may serve as valuable candidates for subsequent functional studies.

However, there are some limitations in this study. First, the potential bias associated with participant overlap can be minimized through the utilization of the F statistic (Pierce and Burgess, 2013). Second, due to the absence of stratification based on demographic data such as sex and race in the original study, further subgroup analysis was not feasible. Third, it is worth noting that the number of loci identified in GM GWASs remains limited, potentially resulting in weak instrumental variables for our Mendelian randomization (MR) studies and subsequently reducing their statistical power. Finally, given that a majority of participants in GWASs were of European descent, caution should be exercised when extrapolating these findings to other racial groups.

In conclusion, our study presents a comprehensive validation of the causal associations between the gut microbiota and AIS, lumbar spondylolisthesis, and spinal stenosis. Specifically, 6 GM taxa were identified to have a positive causal relationship with AIS, whereas 3 GM taxa exhibited a negative causal association. For lumbar spondylolisthesis, 8 GM taxa showed a positive causal relationship, and 6 gut microbiota taxa showed a negative causal relationship. Moreover, 4 GM taxa were positively causally related to spinal stenosis. All these findings suggest the potential utility of these strains as novel biomarkers, providing insights for the treatment and prevention of spinal stenosis diseases.

## Data Availability

The exposure data presented in the study are deposited in the MiBioGen Consortium repository, accession number www.mibiogen.org. The outcome data presented in the study are deposited in the MiBioGen Consortium repository, accession number www.finngen.fi/en. We confirm that the data have been successfully deposited.
